# The whole is greater than the sum of the parts: Recognising missed opportunities for an optimal response to the rapidly maturing TB-HIV co-epidemic in South Africa

**DOI:** 10.1186/1471-2458-9-243

**Published:** 2009-07-16

**Authors:** Rubeshan Perumal, Nesri Padayatchi, Ellen Stiefvater

**Affiliations:** 1 Centre for the AIDS Programme of Research in South Africa (CAPRISA), University of KwaZulu-Natal, Durban, South Africa; 2 Department of Public Health, University of KwaZulu-Natal, Durban, South Africa; 3 Mailman School of Public Health, Columbia University, New York, USA

## Abstract

**Background:**

Despite widely acknowledged WHO guidelines for the integration of TB and HIV services, heavily burdened countries have been slow to implement these and thus significant missed opportunities have arisen.

**Discussion:**

The individual-centred, rights-based paradigm of the SA National AIDS Policy, remains dissonant with the compelling public-health approach of TB control. The existence of independent and disconnected TB and HIV services results in a wastage of scarce health resources, an increased burden on patients' time and finances, and ignores evidence of patients' preference for an integrated service. The current situation translates into a web of unacceptable, ongoing missed opportunities such as failure to maximize collaborative disease surveillance, VCT, adherence support, infection control, and positive prevention. TB services present a readily identifiable cohort for HIV provider-initiated testing. Integrating HAART and DOTS will promote efficient usage of health workers' time and a more navigable experience for patients, ultimately ensuring increased TB treatment completion rates and MDR-TB prevention. As direct observation evolves into a more supportive, empowering experience for patients, adherence to both TB drugs and HAART will be bolstered. Little attention has been paid to the transmission of TB within HIV services. Low cost infection control interventions include: triaging patients, scheduling new and follow-up patients separately; well-ventilated, sheltered waiting rooms; and the use of personal respirators by patients and staff. A more patient-centred approach to TB care may be able to recruit the active participation of TB patients in positive prevention efforts, including maximizing personal infection control, limiting exposure of social contacts to TB during the intensive phase of treatment, advocating isoniazid prophylaxis within the home and patient-centred education efforts to reduce overall transmission. Several model programmes demonstrated synergy, in which the impact of the "whole" or integrated response was greater than the sum of the non-integrated parts.

**Summary:**

The full potential of an integrated TB-HIV service has not been fully harvested. Missed opportunities discount existing efforts in both programmes, will perpetuate the burden of disease, and prevent major gains in future interventions. This paper outlines simple, readily-implementable strategies to narrow the gap and reclaim existing missed opportunities.

## Background

The devastating interaction between the human immunodeficiency virus (HIV) and tuberculosis (TB) has become increasingly reflected in the scientific literature. HIV has resurrected TB as a global health concern, while TB has amplified the mortality from HIV substantially[1]. Negative outcomes for co-infected individuals and disconnected, inadequate services which fail to optimally deal with both epidemics are particularly evident in resource limited settings[2]. In recognition of the deleterious interaction between these diseases, the World Health Organisation(WHO) has developed guidelines for the collaboration of both modalities of care in the pursuit of a coherent, responsive and adequate model[3]. Despite the growing evidence base and the WHO's resounding calls for a stronger connection between TB and HIV care, many of the hardest hit countries have been slow to implement these recommendations on a wide scale[4]. The current situation translates into a web of unacceptable, ongoing missed opportunities such as failure to maximize HIV counselling and testing, collaborative disease surveillance, adherence support, infection control, and positive prevention (Table 1).

**Table 1:** Key missed opportunities in TB/HIV

1. Counselling and testing	
2. Surveillance	
3. Adherence support	
4. Infection control	
5. Positive prevention	

KwaZulu-Natal(KZN), home to nearly 10 million people, is one of the poorest provinces in South Africa and is at the epicentre of the HIV and TB pandemics. In 2006, the incidence of TB in KZN was 1076/100000[5], the HIV prevalence was 39.1%[6] and 80% of TB patients were co-infected with HIV[7]. HIV has resurrected the TB epidemic over the past decade and threatens the already weak provincial health system, in which some facilities have a TB directly observed therapy short-course(DOTS) coverage of less than 30% and others have no TB DOTS programme at all[8]. We share our experiences, from studying the HIV-TB interaction in an urban TB clinic and a specialist TB hospital, on pragmatic strategies for successfully integrating HIV and TB care in the public sector, and highlight significant existing missed opportunities in TB/HIV. The clinic is located in the central business district adjacent to a transportation nodal point, and adjoining an HIV research clinic and treatment programme. Each year approximately 9000 new patients are diagnosed with TB at the clinic; 91 percent of whom are black, 61% male and 53% unemployed. The specialist TB hospital is a public sector specialist referral hospital for the management of TB in KZN, within the South African district health system. There are currently 160 beds, with the inpatient population almost exclusively(97%) comprising patients infected with drug resistant tuberculosis.

Despite an HIV-TB co-infection rate of approximately 75% in some settings[9], the SA National TB Control Programme (SANTCP) and HIV/AIDS control efforts remain largely separate. The transformation of the TB landscape by HIV, demands a public health response that extends well beyond the traditional boundaries of TB control. Similarly, HIV cannot be adequately managed if its intimate relationship with TB is not reflected in HIV services. The public health response to HIV has focused on individuals and safely guarded their right to confidentiality. This inclination toward the rights of the individual with minimal focus on the competing rights of the community is maintained throughout the spectrum of HIV care. The response to TB has always been quite different with the historical response including many restrictions to personal rights. Patients were "institutionalized" and subjected to the necessary treatment in a seemingly paternalistic manner. While much has changed in terms of aligning the response to TB with human rights, the response still reflects an interest in the competing rights of the public, and the need to prevent spread of infection. Programmes have been targeted at communities, and little effort has been made to protect the individual. While TB programmes have benefited from initiatives such as care support buddies and tracing of defaulters, non-disclosure in HIV presents a barrier to treatment adherence. Moreover, it is difficult to shape a public health policy for HIV care with an HIV programme that is individual-centred. The individual-centred, rights-based paradigm, which characterizes much of the SA National AIDS Policy, therefore remains dissonant with the compelling public-health approach of TB control.

The high HIV prevalence in TB patients make these patients and health centres a readily identifiable cohort and facility for efficiently providing HIV counselling, testing and entry into the continuum of HIV care services. Moreover, the high prevalence of HIV in TB suspects strengthens the call for an integrated TB-HIV service[10]. The sustainable and pragmatic solution to the dual epidemics of HIV and TB in sub-Saharan Africa is not as simple as the expansion of either Highly Active Antiretroviral Therapy(HAART) or the TB DOTS strategy independently.

The growing body of scientific literature remains ambivalent on the impact of successful HAART programmes on the burden of TB [11-13]. Factors which are believed to limit this impact include: incomplete TB risk reduction during HAART, late initiation of HAART, low community HAART coverage, and increased life expectancy which extends the period during which HAART patients may develop TB [11,14]. The ambivalence surrounding the impact of HAART programmes in South Africa on the burden of TB may also allude to the importance of eliminating the disjointedness of existing TB and HIV services, in order to maximise the role that HAART may play in reducing community risk of TB[11,12].

The existence of independent and mutually disconnected TB and HIV care systems results in a wastage of scarce health sector resources, an increased burden on patients' time and finances, and ignores evidence of patients' preference to attend an integrated service[10,15]. More importantly, the current situation translates into a web of unacceptable, ongoing missed opportunities. Lessons learned during the past 5 years of HAART scale-up could be applied to the older and less robust TB system, increasing efficiency and effectiveness while simultaneously integrating responses. However, it must be noted that both systems have much to learn from the other, and it is naïve to believe that the TB system is the only beneficiary of a more intimately connected TB-HIV service. TB programmes have decades of experience with standardised treatment procedures, including recording and reporting, which will be invaluable to a partnership with the HIV programme[16,17]. Several model programmes demonstrated synergy, in which the impact and quality of the "whole" or integrated response was greater than the sum of the non-integrated parts[18]. In contrast, non-integration results in less than optimal care for both patient groups. While some have cited change within public health systems as complicated and expectedly gradual, Stephen Lewis former special envoy for HIV/AIDS in Africa, has called the sluggish response "almost criminal"[19]. While we acknowledge that integrated services are challenging to implement in our setting due to the established vertical approach to TB and HIV care, huge patient loads and significant staff shortages, it is crucial to identify and minimise existing missed opportunities if we are to change the trajectory of the dual epidemics.

Attempts to maximize TB interventions will be undermined without an assessment of the profile of patients who present to TB facilities. The South African TB services have largely been informed by the epidemiological history of the disease. Key gender and age assumptions significantly impacted the design and delivery of the services. Almost 70% of all TB patients at our TB facility are now under the age of 45, and almost 40% of all patients are female[20]. Even globally, women account for approximately 36% of smear-positive cases[21]. Despite the substantial evolution of the disease, particularly under the influence of HIV in the developing world, health services have failed to appreciate and respond to the changes, let alone seize the new opportunities presented by the changing profile of patients. Challenging the underlying demographic assumptions which inform the design and delivery of existing TB programmes, such as the belief that TB is primarily a disease of men, will also be necessary if we are to respond to the epidemic appropriately[1]. As TB and HIV services become more intimately connected, there will be an increasing demand for services to be responsively designed for the patient population profile.

## Discussion

### Guiding Principles

Two fundamental principles should guide efforts to construct an integrated complementary response. Firstly, the opportunity presented by the continued scale-up of comprehensive HIV/AIDS care including HAART to simultaneously scale-up the integration of TB and HIV services should be fully exploited. Secondly, scientific knowledge and operational experience gained must diffuse freely between the methodologically distinct TB and HIV services. Scientific and practical knowledge generated since the discovery of HIV 25 years ago should permeate into existing approaches to TB. Conversely, expanding the public health approach of TB control efforts to the patient-centred, rights based approach of HIV/AIDS control is necessary if we are to maximise the impact of a synergised programme.

### Areas of focus

Several points along the continuum of comprehensive care present opportunities for applying the lessons learned between HIV and TB services (Table 1):

With respect to **HIV counselling and testing**, TB services present a readily identifiable cohort for HIV provider-initiated testing (PIT). Service integration in this cohort will undoubtedly increase HIV screening for this high risk cohort. Studies in other African countries have shown that patients are willing to attend integrated services and that, if provided, uptake of voluntary counselling and testing(VCT) or PIT is high[15,22]. Given the 15% annual risk of developing TB in HIV infected individuals[4], a more intimate connection between TB and HIV services would aid early diagnosis and management of TB. PIT in TB clinics can focus on the clinical requirement to know a patient's status in order to provide appropriate medical care[23]. In addition to PIT, point of care CD4 count testing can be conducted to minimise the delay between HIV testing and enrolment into HIV care. The proportion of notified TB patients who have a known HIV status at the end of their outpatient TB treatment at our urban TB clinic using conventional VCT was 21.7% in 2007, having increased from 8.8% in 2004 (p < 0.0001) (Table 2). Amongst patients admitted for treatment of multi-drug resistant(MDR) TB, 51.5% of patients had a known HIV status in 2003, having decreased from 65.7% in 2000 (p < 0.002) (Table 3). It is likely that these patients would have had multiple encounters with health care providers before and during admission, yet the proportion of patients with a known HIV status remains unacceptably low. With the expansion of outpatient VCT facilities in many hospitals and an emphasis on privacy and confidentiality, the wards may increasingly be viewed as unsuitable for VCT. In addition, dually infected inpatients may be perceived to be too ill for VCT or unprepared to deal with a second diagnosis. Given the ambivalence regarding the timing of HAART initiation in TB patients, clinicians may not see the urgency in establishing an HIV diagnosis in an ill TB patient. While randomized studies examining the timing of HAART initiation in TB patients are still underway, preliminary data from our centre suggests a reduction in mortality in excess of 50% if TB treatment and HAART are integrated[24]. The downward trend in the proportion of inpatients that have a known HIV status is concerning, and illustrates a sizable missed opportunity in this setting. A patient-centred approach to VCT in HIV services has evolved successfully. The perceived lack of respect for confidentiality at TB facilities may have rendered it unattractive and unsafe as site for HIV counselling and testing[22]. Combating the stigma associated with HIV, whilst simultaneously preserving respect for confidentiality by ensuring privacy in the setting and communication is essential. To enhance the uptake of counselling and testing, patients in the various waiting areas in our TB clinic receive simple key messages from health care workers on TB and HIV. Initially this intervention was directed at large groups of patients but has evolved into smaller group discussions of 4–6 individuals to improve exchange of information between patients and health care workers. Efficient use of patients' time in the TB waiting area is maximized at our centre by delivering health care messages to a captive audience without interfering with the procedures conducted and flow of patients at the clinic. A similar commitment to patient education has not, however, diffused into the HIV care setting. Rarely, if ever, are formal patient education programmes focused on TB presented to this high risk group. Such programmes may assist the health system in early identification of TB cases by promoting self-reporting by patients.

**Table 2:** HIV status of patients on TB treatment at a TB referral facility in Durban, South Africa

***Year***	***No. of patients seen***	***No. of patients with known HIV status(%)***
2004	8716	770 (8.8%)
2005	8567	1145 (13.4%)
2006	8180	1392 (17.0%)

2007	7202	1564 (21.7%)

P < 0.0001 (Chi-square for trend)

**Table 3:** HIV status of patients on treatment for Multi-Drug Resistant Tuberculosis(MDR-TB) at an MDR referral facility in Durban, South Africa

***Year***	***No. of patients admitted***	***No. of patients with known HIV status(%)***
2000	204	134 (65.7%)
2001	266	159 (59.8%)
2002	384	228 (59.4%)

2003	435	224 (51.5%)

P < 0.002 (Chi-square for trend)

The WHO and UNAIDS have identified TB facilities in settings of high HIV prevalence as important sites for additional HIV **surveillance**. Integrated TB-HIV services will contribute to our knowledge of both epidemics, and facilitate the estimation of the burden of coinfection. As coinfected patients are symptomatic from HIV infection, surveillance at integrated clinics will provide a good estimate of the burden of HIV disease and mortality. This is substantially different from HIV surveillance at antenatal clinics where patients are generally asymptomatic from HIV infection, and only an estimation of population level infection is obtained. Estimates of HIV disease burden in coinfected patients will provide crucial direction to resource allocation and future health care planning. The active monitoring of patients in HIV clinics for the development of TB is an essential element of closer linkages between TB and HIV centres. Routine symptom check lists and clinical cues may be employed at HIV clinics to increase vigilance and identification of TB cases. This will serve as a method of TB case finding in high risk group, as well as give expression to the intimate relationship between TB and HIV to clinicians and patients.

**Adherence support **presents opportunities for enhancing patient support and improving resource utilization. Integrating HAART and the TB DOTS strategy in a manner suitable to the local conditions will initially require investment in health worker training and service reorganization. However, long-term benefits will include efficient usage of health workers' time and a more easily navigable experience for patients, ultimately ensuring increased TB treatment completion rates and MDR-TB prevention. DOT for TB originated almost half a century ago as a method of ensuring adherence to TB drugs and completion of treatment, and is the most successful and well studied of adherence interventions[25]. DOTS programmes for TB could provide the necessary infrastructure through which DOT for HAART could be conducted. As direct observation evolves into a more supportive, empowering experience for patients, adherence to both TB drugs and HAART will be bolstered. This Modified Directly Observed Therapy(in which not all medication doses are administered under direct observation) may also present an opportunity for more closely monitoring adherence to HAART. A pilot study at our TB clinic revealed that a modified DOTS approach, in which the administration of TB medication and HAART was directly observed on weekdays and self-administered on weekends, resulted in a well-tolerated, acceptable and therapeutically beneficial treatment programme[25]. Such a programme may provide an initial structured and supervised HAART experience from which transition may be made toward greater self-administration. Currently at the adjoining HIV clinic, patients self-administer their HAART and receive adherence counselling at five strategic points in their care. These sessions are used to familiarize patients with their diagnosis, the drug therapy they will be exposed to, relevant time-frames, the importance of adherence, and the consequences of non-adherence. These interactions with patients focus on empowering patients and promoting autonomy and commitment to treatment, while emphasising the benefits of adherence. These patients are also counselled on the risk of acquiring TB as well as the symptoms they should be vigilant for. At the TB clinic, patients receive minimal counselling, and few are familiar with their treatment plan or the consequences of non-adherence. In a health systems assessment of KwaZulu Natal, it was found that merely 29% of TB patients knew that their full course of treatments should be completed, and only 2% knew that TB had to be treated for a minimum of 6 months[8]. These lessons from ART should be integrated into TB services to improve understanding of and adherence to treatment, stem the high TB treatment interruption rates and sufficiently educate patients in protecting themselves from re-infection and infecting others. An often neglected element in improving adherence is the commitment and attitude of health care workers who may not necessarily be a part of the formal adherence support programme. A supportive, sensitive, respectful and encouraging attitude from all healthcare workers may promote adherence by making clinic visits more pleasant[8]. The growing call for increased community participation and patient-support as part of the STOP TB Strategy, can be gainfully directed to a less imposing and more empowering treatment programme[4]. This approach will be more respecting of patients' rights to dignity and choice, as set out in the The Patients' Charter for Tuberculosis Care[4]. A single strategy for bolstering adherence to both TB and ARV therapies makes more programmatic sense, and will be more logical and practical to patients, who, unlike the healthcare system, do not compartmentalize their illnesses. A less fragmented approach will advance the paradigm of 'treating the patient and not just the disease'.

Until recently, little attention has been paid to TB **infection control **measures in the developing world. The growing evidence of nosocomial transmission of the infection in both inpatient and outpatient settings has failed to arouse a compelling attempt to address the issue[26,27]. In the era of the dual epidemics of TB and HIV, even less attention has been paid to the transmission of TB within HIV facilities. The risk of TB transmission to health care workers and other HIV infected patients is an understandable challenge to HIV services which articulate intimately with TB services[26]. As a result of the increased risk of negative acid-fast bacilli sputum-smear results in TB-HIV coinfected individuals, there is often a delay in the diagnosis of TB, with a consequently increased exposure to other HIV service users and health care workers[28]. The contribution of smear negative TB to ongoing transmission may have been underestimated, particularly in terms of transmission to immuno-compromised contacts[28]. TB infection control measures in HIV services is based on a three level hierarchy of controls, including administrative or workplace controls, environmental controls, and personal respiratory protection[2]. Low cost interventions include proper and innovative triaging of patients, with expeditious attention to coughing patients. Preventing re-infection of patients in the continuation phase of treatment may include scheduling new and follow-up patients on separate days. Open, well-ventilated, sheltered waiting rooms may be a simple alternative to more sophisticated, resource requiring infrastructure. Without making structural changes to our TB clinic, large waiting areas have been redesigned in favour of multiple smaller waiting areas with open air cover. In addition, a separate well ventilated area is used for known cases of drug resistant TB. Existing structures in established primary health care clinics, at which the majority of TB patients receive care, can be economically modified to improve infection control. In a model of nosocomial transmission of extensively drug-resistant TB in this setting, it was found that improvements to natural ventilation could prevent 33% (8–35%, 95% CI) of future XDR-TB cases[27]. Personal respiratory protection is usually uncomfortable and when made available is ineffectively used in most health settings[29]. Nonetheless, all patients are requested to use surgical masks, while health care workers are provided with personal respirators. However, there is currently no assessment of air quality changes, and staff often complain of a poor face seal of the masks as well as the discomfort of using the mask during the high temperatures experienced in this part of the world. While TB among health care workers constitutes a relatively small proportion of all TB cases, the criticality of staff availability and occupational safety warrants our attention. Promoting HIV testing among health care workers, redeploying HIV infected workers away from the TB unit and ensuring strict adherence to mask use may prevent more than two thirds of staff XDR-TB infections, and possibly offer similar benefits in drug sensitive TB[27]. In addition, early diagnosis and treatment of TB as would be possible in an integrated service would in itself represent a public health infection control effort. Isoniazid prophylaxis remains an underutilized measure to reduce TB transmission in HIV services, especially among patients with a past history of TB[2,30].

Finally, HIV has propelled the innovative approach of **positive prevention **for intervening in both the transmission of infection and the development of illness. The strong prevention arm of HIV programmes is only vaguely present in the TB programme, and is usually confined to isoniazid prophylaxis for contacts of TB patients. Key lessons and opportunities for positive prevention in TB have not been fully exploited. A more patient-centred approach to TB care may be able to recruit the active participation of TB patients in positive prevention efforts, which could include maximizing personal infection control, limiting exposure of social contacts to TB during the intensive phase of treatment, advocating isoniazid prophylaxis within the home and patient-centred education efforts to reduce overall transmission. Disclosure of HIV status remains a significant challenge to HIV prevention. While a pilot study at our clinic revealed that approximately two thirds of patients disclosed their status to one other person with the hope of receiving support from them, only few patients disclosed it to their sexual partner[31]. The common law of South Africa places an ethical and moral duty on the health system to inform vulnerable uninfected partners of TB and HIV infected patients, after impressing upon the patient the importance of disclosure to their sexual partners. Importantly, these partners have a reciprocal moral and ethical duty to receive such information. Partner notification for HIV may subtly and opportunistically piggy-back on contact tracing efforts in TB. Even without this, contacts of TB who present to health services in response to TB contact tracing can be considered a high-risk, readily identifiable cohort for HIV testing. Every patient, therefore, serves as an entry point to a network of vulnerable individuals[17]. The assessment of vulnerability in our context is a complex issue and undoubtedly requires a thorough evaluation of the pros and cons of facilitating such a disclosure to sexual partners of the patient.

## Summary

The deleterious interaction between the dual epidemics of TB and HIV has crippled the health systems of many developing countries. While significant gains have been made in the last decade, including the roll-out of HAART and the implementation of the DOTS Strategy, many lessons are yet to be learned. Perhaps, the greatest consequences will arise, not from what has been done, but rather from what has been failed to be done. Indeed, TB and HIV have presented many challenges independently and through their interaction, but their close association has also created fertile ground for cross-pollination of knowledge and ideas between the historically distinct scientific communities and health programmes. The result is a wasteful failure to seize the opportunities presented by the intimate intertwining of the TB and HIV epidemics.

## Competing interests

The authors declare that they have no competing interests.

## Authors' contributions

All authors contributed to conceptualising and writing up the research.

## Pre-publication history

The pre-publication history for this paper can be accessed here:


